# Testing the efficacy and efficiency of a single “universal warming protocol” for vitrified human embryos: prospective randomized controlled trial and retrospective longitudinal cohort study

**DOI:** 10.1007/s10815-018-1276-4

**Published:** 2018-08-03

**Authors:** L. Parmegiani, K. H. Beilby, A. Arnone, S. Bernardi, A. M. Maccarini, E. Nardi, G. E. Cognigni, M. Filicori

**Affiliations:** 1 Reproductive Medicine Unit- GynePro Medical Centers GynePro Medical, Via T. Cremona, 8-40137, Bologna, Italy; 2 Department of Obstetrics & Gynaecology, Monash University – Melbourne, Melbourne, Australia; 3 Department of Medical and Surgical Sciences, University of Bologna, Bologna, Italy

**Keywords:** Universal warming procedure, Vitrification, Warming, Embryo cryopreservation, Kitazato Vitrification Kit, Sage Vitrification Kit

## Abstract

**Purpose:**

To study the efficacy and efficiency of a “universal warming protocol” for vitrified human embryos, based on subsequent steps with 1 and 0.5 M concentration of extracellular cryoprotectant (ECCP).

**Method:**

Two studies on patients undergoing fertility treatments via ICSI: a prospective randomized controlled trial (RCT) and a retrospective cohort study (CS). Setting: Private assisted reproductive (AR) center.

**RCT**: duration 01/03/2017–01/10/2017; 315 embryos at blastocyst stage obtained from 169 patients. Each patient’s embryos were first randomized for vitrification with two different kits: Vitrification Kit (Kitazato, Japan) and Sage Vitrification Kit (Origio, Denmark). The embryos were randomly warmed with either Kitazato (K) or Sage (S) warming kits, specifically: group A (KK), group B (KS), group C (SK), and group D (SS). Primary outcome measure: survival rate (number of embryos surviving per number of embryos warmed). Secondary: implantation rate (number of embryos implanted per number of embryos transferred).

**CS**: duration 01/01/2013–31/12/2015 embryos from patients’ own oocytes; 10/04/2015–31/07/2017 embryos from donors’ oocytes. A total of 1055 embryos vitrified at cleavage stage obtained from 631 warming cycles: 847 of these obtained from patients’ own oocytes, 208 egg-donation-derived embryos. Each patient’s embryos were vitrified and warmed in various combinations of three different vitrification/warming kits: Kitazato (K), Sage (S), or made in-house in our laboratory (H). Vitrification/warming kits from different manufacturers are routinely used in our AR center, and the warming procedures are randomly performed with any available kit on a “first-in-first-out” basis, irrespective of the kit used for vitrification. Group names: KK, KS, SK, SS, SH, HK, HS, HH (embryos from patients’ own oocytes); eKK, eKS, eSK, eSS (egg-donation-derived embryos).

**Results:**

Cryo-survival rates were comparable in all study groups.

**RCT**. Group A 99.0% (96/97), group B 98.8% (83/84), group C 98.4% (61/62), and group D 98.6% (71/72).

**CS**. Embryos from patients’ own oocytes: KK 96.4% (54/56), KS 100.0% (13/13), SK 98.8% (80/81), SS 97.2% (174/179), SH 97.6% (40/41), HK 95.2% (20/21), HS 99.5% (187/188), and HH 97.4% (261/268). Egg-donation-derived embryos: eKK 100.0% (91/91), eKS 98.4% (60/61), eSK 100.0% (26/26), and eSS 96.7 (29/30).

Implantation was generally comparable in all study groups—exceptions were in CS: KS vs. SK (*P* = 0.049), SS (*P* = 0.012), HS (*P* = 0.010), HH (*P* = 0.025); and SH vs. SS (*P* = 0.042), HS (*P* = 0.035).

**Conclusion:**

Worldwide, millions of embryos have been cryopreserved using different vitrification kits; these studies establish that it is possible to combine different kits for vitrification and warming using a universal warming protocol. This can optimize costs, simplify lab routines, and favor embryo exchange between IVF centers.

**RCT registration number:**

ISRCTN12342851.

## Introduction

In assisted reproductive (AR) laboratories, human oocytes, embryos, and ovarian tissue have been cryopreserved over the past decades by two main methods: vitrification (VIT) or slow freezing (SF) [1, 2]. Because of its guarantee of high survival rates, the use of VIT has overtaken SF [2, 3]. Nevertheless, oocytes, embryos, and ovarian tissue that have been slow frozen are still stored in countless cryobanks worldwide. Furthermore, AR centers may receive oocytes, embryos, and tissue transported from other centers, cryopreserved with various different SF and VIT protocols. Since regulations recommend the use of FDA/CE marked warming media approved for human AR and because the shelf life of these media is usually short (some months), it is expensive to keep available the reciprocal solution for every manufacturers’ cryopreservation kit. In this scenario, the possibility of using a “universal medium” to warm any cell or tissue irrespective of the freezing protocol may simplify the management of warming procedures [4].

In a previous basic research study, we already demonstrated that it is possible to warm slow frozen human oocytes by using a single “universal warming protocol” based on subsequent steps with 1 and 0.5 M concentration of extracellular cryoprotectant (ECCP) [4]. The efficacy of this “universal warming” was subsequently confirmed by a multicenter study performed on 400 slow frozen oocytes [5, 6]. Since the oocyte is the most sensitive human reproductive cell to cryoinjury, our pilot studies potentially paved the way for the clinical use of this single warming protocol for any reproductive cell, irrespective of the cryopreservation method used for freezing. Today, millions of embryos are vitrified worldwide using the different kits available on the market; these ready-to-use VIT kits contain vitrification/warming (VIT/WARM) solutions with only slight differences in their composition. Although we may hypothesize that combining different VIT/WARM kits is feasible, it nevertheless remained to be demonstrated that a warming solution of one manufacturer can be used to warm the embryos vitrified with another kit. The aim of the present study is to assess for the first time the clinical efficacy and efficiency of a universal warming protocol on vitrified embryos by analyzing the cryosurvival and implantation rates obtained by combining different VIT/WARM kits having 1 and 0.5 M of ECCP in the warming solutions.

## Materials and methods

### Study design

Two studies on infertile patients undergoing fertility treatments via ICSI were performed in our private assisted reproductive (AR) center: a prospective randomized controlled trial (RCT) and a retrospective cohort study (CS). The total number of vitrified/warmed embryos studied was 1370 (315 for RCT1055 for CS). All the women participating in our cryopreservation program were informed about the procedure and gave their written consent; the study was approved by the Institutional Review Board of the clinic (approval number 22.02.2017).

### Randomized controlled trial (RCT)

**Fig. 1:**
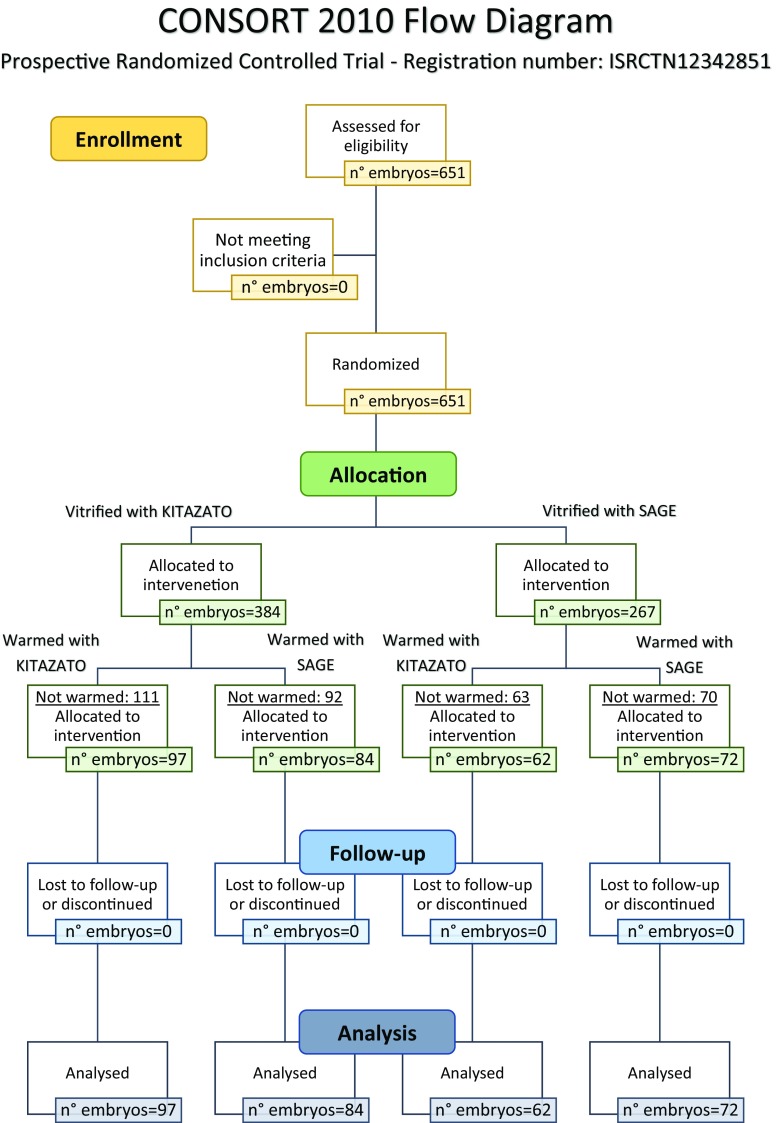
Randomized controlled trial, CONSORT flowchart

This prospective randomized study was performed on 315 embryos at the blastocyst stage obtained from 205 warming transfer cycles performed on 169 patients (Fig. 1). Each patient’s embryos were first randomized for vitrification (VIT) with two different kits: Vitrification Kit (Kitazato, Japan) and Sage Vitrification Kit (Origio, Denmark). At warming, the embryos were randomly allocated to either the Kitazato or the Sage warming kit, specifically:

- Group A—97 embryos vitrified with Kitazato and warmed with Kitazato
- Group B—84 embryos vitrified with Kitazato and warmed with Sage
- Group C—62 embryos vitrified with Sage and warmed with Kitazato
- Group D—72 embryos vitrified with Sage and warmed with Sage

Randomization was performed by using a specific software tool (http://www.randomizer.org). Inclusion criteria were female age at freezing ≤ 42 and embryos not biopsied for preimplantation genetic test (PGT). Cleavage stage embryos and egg-donation-derived embryos were excluded. The primary endpoint was survival rate (number of embryos surviving per number of embryos warmed). The secondary endpoint was implantation rate (number of gestational sacs at ultrasound examination per number of embryos transferred). See “Statistical analysis” section for sample size calculation. The duration of the study was 01/03/2017–01/10/2017. This was not a double-blind study; for safety and regulatory compliance, it is forbidden to blind embryologists to the labels and manufacturers of media used in AR laboratories. The patients and their physicians were blinded. Trial registration number: ISRCTN12342851 (10.1186/ISRCTN12342851).

### Cohort study (CS)

This retrospective observational study was performed on 1055 embryos vitrified at cleavage stage obtained from 631 warming transfer cycles: 847 embryos were obtained from patients’ own oocytes (498 warmings), whereas 208 (133 warmings) were egg-donation-derived embryos. In this study, each patient’s embryos were vitrified and warmed in various combinations of three different VIT/WARM kits: Kitazato (K), Sage (S), and made in-house in our laboratory (H). In our AR center, VIT/WARM kits from different manufacturers are routinely used (to avoid dependence on a single supplier and potential problems in stock delivery) and the warming procedures are randomly performed with any available kit on a “first-in-first-out” basis (kits with earliest expiration date are used first), irrespective of the kit used for VIT. More specifically:

- Group KK—56 embryos vitrified with Kitazato and warmed with Kitazato
- Group KS—13 embryos vitrified with Kitazato and warmed with Sage
- Group SK—81 embryos vitrified with Sage and warmed with Kitazato
- Group SS—179 embryos vitrified with Sage and warmed with Sage
- Group SH—41 embryos vitrified with Sage and warmed with in-house kit
- Group HK—21 embryos vitrified with in-house kit and warmed with Kitazato
- Group HS—188 embryos vitrified with in-house kit and warmed with Sage
- Group HH—268 embryos vitrified with in-house kit and warmed with in-house kit

By chance, no embryos were vitrified with Kitazato and warmed with the in-house kit.

In the case of egg-donation-derived embryos, the study groups were:

- Group eKK—91 embryos vitrified with Kitazato and warmed with Kitazato
- Group eKS—61 embryos vitrified with Kitazato and warmed with Sage
- Group eSK—26 embryos vitrified with Sage and warmed with Kitazato
- Group eSS—30 embryos vitrified with Sage and warmed with Sage

Embryos from patients’ own oocytes were warmed between 01/01/2013 and 31/12/2015, and embryos from donor oocytes between 10/04/2015 and 31/07/2017.

### Controlled ovarian stimulation, oocyte retrieval, ICSI, and embryo culture

Controlled ovarian stimulation, transvaginal ultrasound-guided oocyte retrieval, oocyte decumulation, ICSI, and embryo culture were performed as previously described by our group [7, 8]. Embryo culture at low oxygen tension (5%) was performed in Embryoscope or Embryoscope + (Vitrolife, Sweden) for RTC, and in BT37 Planer (Origio) for CS. The embryos not transferred during the fresh cycle were vitrified, and their performance at warming is analyzed in this study.

### Donor oocytes

Vitrified oocytes from young donors were obtained due to a cooperation agreement between our AR center and two gamete cryobanks (Ovobank Marbella Spain and IMER Valencia Spain) as regulated by Italian legislation [9, 10]. The oocytes were transported to our AR center and warmed according to the Kitazato protocol [11, 12]. The warmed oocytes were inseminated by ICSI and cultured as elsewhere described [1]. The embryos not transferred during the oocyte warming cycle were vitrified, and their performance at warming is analyzed in this study.

### Vitrification/warming kits

Kitazato vitrification and warming solutions contain trehalose as ECCP and are supplemented with hydroxypropyl cellulose (HPC) [13]. Sage and in-house kits contain sucrose as ECCP and are supplemented with human serum albumin (HSA) [1, 14]. The basic medium is TCM199 for Kitazato, modified HTF with MOPS for sage and PBS (D8662 Sigma-Aldrich, Italy) for home-made kits [1, 11, 12, 14]. The cryoprotectant cocktail comprises 7.5% dimethylsulfoxide (DMSO)-7.5% ethylene glycole (EG) in equilibration solution, and 15% DMSO-15% EG in vitrification solution [11] in all the VIT kits. All the warming kits involve sequential steps with 1 and 0.5 M concentration of ECCP in the warming solution.

### Universal Vitrification procedure

Vitrification was performed with Cryotop (Kitazato, Japan) [11, 12] in certified sterile liquid nitrogen (SLN_2_) [15]. SLN_2_ was produced with a specifically designed device, Nterilizer™ as described elsewhere [4, 16]. Each embryo was vitrified following a “universal” protocol that differs slightly from the Instructions for Use (IFU) suggested by the manufacturers of different kits, as described below.

1. The whole procedure was performed at room temperature (20–25 °C) minimizing exposure of specimens to light during incubation in equilibration (ES) and vitrification (VS) solutions.
2. The solutions were brought to room temperature at least 30 min before use, and the contents of each vial of ES and VS were well mixed by gentle inversion several times before use and aseptically dispensed into a six-well multi-dish (OOPW-SW02 Sparmed, Denmark): 300 μL of ES into well 1 and 300 μL of VS into both well 2 and well 3.
3. The embryo was transferred with a minimal volume of culture medium using a pipette with an inner tip diameter of ~ 200 μm (Stripper tips MXL3, Humagen, USA) to the top of well 1 containing ES. (The embryo free-falls in ES within 30 s, and then it shrinks and subsequently gradually re-expands to its original size within 12–15 min, indicating that equilibration is complete; partial re-expansion after 15 min is observed in the case of expanded, hatched, or collapsed blastocysts).
4. Using a new transfer pipette, pre-loaded with VS, the embryo was transferred from the ES well into the center of the first VS well (VS1). (For the first 40 s, the embryo is gently swirled in the VS1 to thoroughly bathe it in the VS solution).
5. After a total of 50 s, the embryo was moved to the second VS well (VS2), with minimal volume from the VS1 well.
6. Finally, the embryo was loaded in < 1 μL droplet of VS solution into the vitrification device and plunged directly in SLN_2_. The amount of time between first placing the embryo in the two VS solutions and immersion into liquid nitrogen did not exceed 120 s.

### Universal warming procedure

The universal warming protocol has slight differences from the IFU suggested by the manufacturers of different kits. The solutions used for embryo warming are as follows: 4 mL of 1 M ECCP warming solution (1 M WS), 300 μL of 0.5 M ECCP warming solution (0.5 M WS), and 300 μL + 300 μL of washing solution (basic medium—see “Vitrification/warming kits” section). The procedure is described below.

1. The first warming step was performed with the 1 M ECCP warming solution at 37 °C. The vial containing this solution was pre-warmed to 37 °C at least 60 min before use and kept closed throughout.
2. The other solutions were brought to room temperature (20–25 °C) at least 30 min before use; the contents of each vial were well mixed by gentle inversion several times before use and aseptically dispensed into a six-well multi-dish (OOPW-SW02 Sparmed, Denmark): 300 μL of 0.5 M into well 1, 300 μL of washing solution into well 2, and another 300 μL into well 3.
3. A pre-warmed petri dish (OOPW-TF03, Sparmed) was then filled with 4 mL of the warmed 1 M ECCP solution.
4. The vitrification carrier device containing the embryo was opened in certified SLN_2_ [4, 16]: The SLN_2_ insulated container was placed close to the stereomicroscope for rapid manipulation.
5. The strip of the vitrification carrier was immediately plunged into the petri dish containing the warmed 1 M solution (the embryo floats from the carrier to the top of the 1 M solution dish and is kept in this solution for 1 min until it starts to shrink).
6. Using a pipette containing some of the 1 M solution, the embryo was transferred from the petri dish to well 1 of 6-well (300 μL of 0.5 M ECCP) for 3 min minimizing exposure to light (the embryo remains shrunken for the whole duration of this step).
7. Then, drawing up some 0.5 M solution from well 1, the embryo was transferred to well 2 (300 μL of washing solution) for 5 min.
8. Finally, the embryo was placed in well 3 for the last wash (1 min) and subsequently moved to a dish of pre-equilibrated culture medium and incubated at 6% CO_2_–5% O_2_ incubator—37 °C for 1–2 h prior to embryo transfer.

### Endometrial preparation and embryo transfer

Preparation of the endometrium for the embryo transfer (ET) was performed as described elsewhere [8]. Embryo transfer was carried out after three (day 3—CS) or 5 days (day 5—RCT) from progesterone administration [17]. Clinical pregnancy was defined as the presence of a gestational sac with or without fetal heart beat (FHB) at ultrasound examination, 2 weeks after positive hCG testing.

### Statistical analysis

Sample size for RCT was calculated to test the kits’ equivalence for the cryo-survival rate (http://clincalc.com/stats/samplesize.aspx). Calculation of sample size was based on our experience with vitrification, assuming the mean survival rate of 98% obtained in our center in the previous 5 years and considering 70% as minimum competence and 95% as benchmark [18]. This analysis revealed that at least 35 embryos would be necessary for each group to obtain a power of 80% and a confidence interval of 95%. The study was closed when all the groups had exceeded the required sample size and when three of the four groups had reached double the minimum number of embryos warmed.

Continuous variables are presented as mean ± standard deviation. Categorical variables are presented as absolute and relative frequencies. Normality of distribution of continuous variables was assessed with a Kolmogorov-Smirnov test (with Lillefor correction). Between-group differences of normally distributed continuous variables were assessed with parametric statistic (Student’s *t* test), whereas non-parametric statistics (Mann-Whitney Rank Sum Test) were employed when the normality test was not passed. Between-group differences in frequencies were assessed using the *χ*^2^ method with Yates correction if needed or Fisher exact test when frequencies were less than 5 in one of the two groups. Data analyses were performed in SPSS Statistics package (version 23, IBM Co., Armonk, NY, USA) and in R 3.4.2. Two-tailed *P* values less than 0.05 were considered significant.

## Results

Female mean age and survival rate were statistically comparable between the study groups (Tables 1, 2, and 3). Mean number of transferred embryos was statistically comparable between the study groups and varied from 1.48 ± 0.08 to 1.57 ± 0.08 in RCT and from 1.68 ± 0.06 to 1.86 ± 0.14 in CS.

**Table 1:** RCT—prospective randomized controlled trial

	Group A	Group B	Group C	Group D
Mean female age (± SD) at freezing	35.5 ± 4.3	35.9 ± 3.8	36.3 ± 4.4	35.5 ± 4.6
No. of surviving embryos/warmed embryos (%)	96/97 (99.0)	83/84 (98.8)	61/62 (98.4)	71/72 (98.6)
No. of embryos implanted/embryos transferred (%)	18/96 (18.8)	15/83 (18.1)	11/61 (18.0)	16/71 (22.5)

*P* value NS

**Table 2:** LCS—retrospective longitudinal cohort study (embryos obtained from patients’ own oocytes)

	Group KK	Group KS	Group SK	Group SS	Group SH	Group HK	Group HS	Group HH
Mean female age (± SD) at freezing	36.0 ± 5.4	35.0 ± 5.1	37.4 ± 3.9	36.6 ± 4.5	36.3 ± 4.6	37.4 ± 3.9	37.0 ± 4.2	36.5 ± 4.3
No. of surviving embryos/warmed embryos (%)	54/56 (96.4)	13/13 (100)	80/81 (98.8)	174/179 (97.2)	40/41 (97.6)	20/21 (95.2)	187/188 (99.5)	261/268 (97.4)
No. of embryos implanted/embryos transferred (%)	11/54 (20.4)	6/13 (46.2)	14/80 (17.5)	24/163 (14.7)	12/40 (30.0)	3/20 (15.0)	26/179 (14.5)	45/260 (17.3)

*P* value NS. Exceptions (implantation rate): 0.049 (KS vs SK), 0.012 (KS vs SS), 0.010 (KS vs HS), 0.025 (KS vs HH), 0.042 (SH vs SS), 0.035 (SH vs HS)

**Table 3:** LCS—retrospective longitudinal cohort study (egg-donation-derived embryos)

	Group eKK	Group eKS	Group eSK	Group eSS
Mean female age (± SD) at freezing	42.5 ± 5.5	38.8 ± 6.3	41.3 ± 4.8	39.5 ± 5.8
No. of surviving embryos/warmed embryos (%)	91/91 (100)	60/61 (98.4)	26/26 (100)	29/30 (96.7)
No. of embryos implanted/transferred (%)	13/91 (14.3)	10/58 (17.2)	4/26 (15.4)	5/29 (17.2)

*P* value NS

### RCT results

In the RCT, cryo-survival rate was group A 99.0% (96/97), group B 98.8% (83/84), group C 98.4% (61/62), and group D 98.6% (71/72). Implantation rates were comparable: group A 18.8% (18/96), group B 18.1% (15/83), group C 18.0% (11/61), and group D 22.5% (16/71) (Table 1).

### CS results

In the CS, cryo-survival rate was group KK 96.4% (54/56), group KS 100.0% (13/13), group SK 98.8% (80/81), group SS 97.2% (174/179), group SH 97.6% (40/41), group HK 95.2% (20/21), group HS 99.5% (187/188), and group HH 97.4% (261/268); implantation was generally comparable in all study groups—exceptions were KS vs. SK, SS, HS, HH; and SH vs. SS, HS (Table 2). In CS with egg-donation-derived embryos, cryo-survival was group eKK 100.0% (91/91), group eKS 98.4% (60/61), group eSK 100.0% (26/26), and group eSS 96.7 (29/30); implantation was comparable between the groups (Table 3). By patients’ request, some of the surviving embryos were cultured until the blastocyst stage before transfer; the failure of blastulation was the cause of the reduced number of embryos available for transfer in some groups: the non-transferred embryo rate was 6.3% (11/174) SS, 4.2% (8/187) HS, 0.4% (1/261) HH, 0.3% (2/60) eKS, and 0% in the other groups. The percentage of embryos transferred at blastocyst stage was statistically comparable between groups: patient-egg-derived embryos 5.5% (3/54) KK, 7.6% (1/13) KS, 5.0% (4/80) SK, 4.9% (8/163) SS, 7.5% (3/40) SH, 5.0% (1/20) HK, 4.4% (8/179) HS, and 4.2 (11/260) HH; and egg-donation-derived embryos 2.2% (2/91) eKK, 0% (0/58) eKS, 3.8% (1/26) eSK, and 3.4% (1/29) eSS. The percentage of transfers with embryos derived from the same patient in the same group (repeated measures) was statistically comparable between groups: 6.8% (2/29) KK, 0% (0/7) KS, 5.0% (2/40) SK, 4.1% (2/97) SS, 8.6% (2/23) SH, 0% (0/14) HK, 5.0% (6/119) HS, 4.5% (8/176) HH, 5.0% (3/60) eKK, 5.0%(2/40) eKS, 0% (0/15) eSK, and 0% (0/18) eSS.

## Discussion

In AR laboratories worldwide, embryos are vitrified using the different kits available on the market; these ready-to-use VIT kits contain VIT/WARM solutions with only slight differences in their composition. Combining different VIT/WARM kits with 1 and 0.5 M of ECCP in the warming solution has been shown to be feasible for oocytes [4–6], but before now, it still remained to be demonstrated that a warming solution of a given manufacturer could be used to warm embryos vitrified with another kit.

The present paper illustrates the clinical efficacy and efficiency of a universal warming protocol with 1 and 0.5 M ECCP for warming on vitrified embryos by analyzing the cryo-survival rate obtained when combining different VIT/WARM kits in two studies: a randomized control trial (RCT) on 315 blastocysts and a cohort study (CS) on 1055 cleavage stage embryos. Survival was statistically comparable in all the groups studied.

### RCT

In the RCT, the blastocysts’ survival rate ranged from 98.4 to 99.0% which is aligned with the benchmark for cryo-Key Performance Indicator (KPI) [18, 19]. Blastocysts were considered to have survived when total of partial re-expansion was observed within 2 h from warming. The secondary outcome measure for the RCT was implantation rate, which was statistically comparable between study groups and ranged from 18.0 to 22.5% and aligns with the fresh implantation rate in our clinic (Parmegiani, unpublished data) and with the value for this KPI [18, 19]. In the RCT, the study population was comparable between groups, and the sample size was calculated in advance; the study was strict, but not double-blinded for the embryologists involved for safety and regulation reasons as explained in “Materials and methods” section. This is the only RCT in the literature investigating the potential combination of VIT/WARM kits from two different manufacturers. The main limitations of this RCT are that it was carried out in a single IVF center and that it was registered before the randomization for warming but after the first randomization for vitrification.

### CS

In the CS, cryo-survival rate was also comparable between all the groups studied, for embryos obtained both from patients’ own oocytes and from donors’, ranging from 96.4 to 100.0%. Embryos were considered to have survived if 100% of the blastomeres were intact at 2 h from warming. Mean female age was comparable between the study groups. Implantation was generally comparable in all the study groups and aligned with the fresh implantation rate in our clinic (Parmegiani, unpublished data) and with the competency value for this KPI [18, 19]. Exceptions were KS vs. SK, SS, HS, HH and SH vs. SS, HS. The higher implantation in KS and SH was probably due to the low number of embryos transferred in these groups and, particularly for KS, also to the lowest mean age (although mean age was not statistically different between groups); it may also be accounted for by the slightly higher rate of embryos transferred at blastocyst stage. The implantation was not affected by repeated measures derived from embryo transfers to the same patient in the same group.

Implantation rate was slightly lower in the groups with embryos generated from donor oocytes; this may be related to the fact that in these patients, all the cryo-transfers were performed after an unsuccessful previous first transfer with fresh embryos. In our center’s egg donation program, implantation rate at first transfer is 37% (Parmegiani, unpublished data). This program started in 2015, and so far, none of the patients who obtained a baby after first transfer has returned to receive a frozen embryo transfer (FET). In contrast, the patients in groups with donor oocytes were all at their second transfer and may therefore have had poor prognosis and low implantation rate at FET. This observation is in line with that reported by other authors investigating cryo-transfer from cryopreserved oocytes or after a failed first transfer [20, 21]. It should also be pointed out that this is the only study in the literature giving information about the cryo-transfer of embryos obtained from vitrified donor oocytes in a transnational egg donation program in Europe. Since it has been demonstrated that the handling necessary for oocyte shipping can adversely affect their survival rate, this may be another factor that could potentially negatively affect the derived embryos [9]. This CS is the first study to compare the effect of different combinations of three VIT/WARM solutions and can be considered a large-scale investigation because it was performed on over a thousand cryopreserved embryos. This study’s limitations are that it was performed in a single IVF center and that it is a retrospective observational study.

### Other limitations

In both RCT and CS, embryo selection—based on morphology or morphokinetics—was not performed before vitrification in accordance with the Italian regulation which prohibits the discarding of embryos with the exception of those with clear defects in fertilization or evolution. Furthermore, it has not yet been possible to assess live birth rates or neonatal outcomes, and this should be addressed in future studies.

### Comments

This double study (RCT and CS) describes the clinical application on embryos of the universal warming protocol, previously tested only on slow frozen oocytes [4]. After that basic science study, which demonstrated that it was feasible to warm human oocytes with a 1–0.5 M ECCP universal procedure irrespective of the freezing protocol, we confirmed the reproducibility of this procedure with a multicenter study [5, 6]. The question which remained to be answered after these first pilot studies was the clinical efficiency of this warming procedure on oocytes and embryos. A 2017 study [22] investigated the survival rate of 79 slow frozen embryos derived from abnormal fertilization (3 pronuclear (3PN)): The survival rate after rapid warming following Parmegiani’s described procedure was 88.6% (70/79). Furthermore, from November to December 2016, the same authors performed 11 warming cycles on slow frozen embryos derived from normal fertilization and obtained 5 clinical pregnancies. So far, this Chinese study has been the only report of the clinical application of Parmegiani’s universal warming. Now, the present study shows the efficiency of a universal warming procedure based on 1–0.5 M ECCP on human embryos in a systematic way. Thus, after the breakthrough in 2014 describing warming performed irrespective of freezing protocol [4], this demonstration of the clinical efficiency of a universal warming procedure may represent a further milestone in human oocyte/embryo cryopreservation and provide guidelines for clinical practice. In addition, the way is paved for future studies to confirm the clinical efficiency of this universal protocol on embryos or oocytes with different combinations of kits from different manufacturers, containing 1–0.5 M of ECCP. In the present study, we compared kits with sucrose and trehalose as ECCP, supplemented by human albumin or hydroxypropyl cellulose. Sucrose is the most commonly used ECCP in cryopreservation protocols; disaccharide trehalose is employed by certain species to survive in extreme conditions [23] and is used as an osmotic agent in some human and mouse cryopreservation protocols [24]. Hydroxypropyl cellulose (HPC) is a replacement for human albumin (HA) or serum substitute supplement (SSS) for use in cryoprotectant solutions to protect embryos/oocytes against injury during vitrification; the current trend towards removing any component of human origin has led to the development of formulations for vitrification kits that are free of viral contamination risk and plasma derivatives [13]. The combination of HPC and trehalose has been used by some manufacturers of vitrification kits to satisfy the requirements of different consumers [25].

## Conclusions

In the last decade, oocytes and embryos have been cryopreserved using vitrification kits from different manufacturers; with this paper, we demonstrate systematically the clinical feasibility of combining different kits for vitrification and warming. Despite limitations to these two studies (RCT and CS) individually, taken together, they support the safe and efficacious use of a universal warming protocol and provide reassurance about safely combining different vitrification and warming solutions.

Since the range of products on the market, and their components’ different origins can sometimes cause confusion for operators regarding how best to achieve good results, this universal warming protocol based on 1 and 0.5 M of ECCP permits efficient warming of vitrified embryos, irrespective of the freezing kit’s manufacturer and of its cryoprotectants and basic medium. The protocol may also potentially be clinically applied to oocytes and ovarian tissue, after appropriate studies. This can optimize costs, simplify lab routines, and favor oocyte/embryo exchange between IVF centers.
